# Evaluation of preoperative visual pathway impairment in patients with non-functioning pituitary adenoma using diffusion tensor imaging coupled with optical coherence tomography

**DOI:** 10.3389/fnins.2023.1057781

**Published:** 2023-02-09

**Authors:** Yanhua Pang, Zhi Tan, Xinxin Chen, Zhihui Liao, Xin Yang, Qin Zhong, Baqi Huang, Qianshuo Zhong, Jingxiang Zhong, Wei Mo

**Affiliations:** ^1^Department of Ophthalmology, Affiliated Hospital of Guangdong Medical University, Zhanjiang, Guangdong, China; ^2^Department of Radiology, Affiliated Hospital of Guangdong Medical University, Zhanjiang, Guangdong, China; ^3^Teaching and Research Center of Medical Communication Science, Affiliated Hospital of Guangdong Medical University, Zhanjiang, Guangdong, China; ^4^Department of Neurosurgery, Affiliated Hospital of Guangdong Medical University, Zhanjiang, Guangdong, China; ^5^Department of Ophthalmology, The First Affiliated Hospital of Jinan University, Guangzhou, China; ^6^Department of Ophthalmology, The Sixth Affiliated Hospital of Jinan University, Guangzhou, China

**Keywords:** non-functioning pituitary adenoma, diffusion tensor imaging (DTI), visual pathway injury, optical coherence tomography (OCT), circumpapillary retinal nerve fiber layer (CP-RNFL)

## Abstract

**Objective:**

Optic chiasma compression and associated visual impairment induced by a non-functioning pituitary adenoma (NFPA) is commonly assessed by the optic disk and retina but is inadequate to understand the entire visual pathway impairment. We aim to evaluate the use of optical coherence tomography (OCT) coupled with diffusion tensor imaging (DTI) for the preoperative evaluation of visual pathway impairment.

**Methods:**

Fifty-three patients with NFPA (categorized into mild and heavy compression subgroups) were subjected to OCT to calculate the thickness of the circumpapillary retinal nerve fiber layer (CP-RNFL), macular ganglion cell complex (GCC), macular ganglion cell layer (GCL), and macular inner plexus layer (IPL), as well as to DTI to calculate the fractional anisotropy (FA) and apparent diffusion coefficient (ADC) values.

**Results:**

Compared to mild compression, heavy compression caused decreased FA value, increased ADC value of several segments of the visual pathway, thin temporal CP-RNFL, and quadrant macular GCC, IPL, and GCL. Average CP-RNFL thickness, inferior-macular inner-ring IPL and GCC thicknesses, inferior CP-RNFL thickness, and superior CP-RNFL thickness were the best indicators of the impairment of the optic nerve, optic chiasma, optic tract, and optic radiation, respectively.

**Conclusion:**

DTI and OCT parameters can effectively evaluate visual pathway impairment and are beneficial for the objective preoperative evaluation of visual pathway impairment in patients with NFPA.

## Introduction

Non-functional pituitary adenoma (NFPA) is the most common type of pituitary adenoma (PA) and is characterized by a lack of hormone-related clinical symptoms and signs (Melmed et al., 2022). Delayed diagnosis of NFPA caused by the absence of indications generates giant adenomas and subsequently extends to the surrounding structures. Compressed optic chiasma and the resulting visual impairment patterns (i.e., vision loss and visual field defects) occur in 78% of patients with NFPA (Subramanian et al., 2021). Ophthalmic examinations and the assessment of visual pathway impairment are critical for early disease diagnosis and surgery decisions for NFPA. Although visual field examination and visual evoked potentials can be used to identify impaired visual fields or pathways, the results are relatively subjective and time-consuming. Given these limitations, many studies have explored more objective and quantitative methods for assessing visual impairment in patients with NFPA.

Optical coherence tomography (OCT) is a new technique for predicting outcomes after the surgical decompression of PA (Chung et al., 2020; Agarwal et al., 2021; Santorini et al., 2022). In addition, diffusion tensor imaging (DTI) is a new type of functional magnetic resonance used for objectively assessing white matter fiber connectivity and integrity in the central system tissue by detecting the diffusion differences between the parallel and vertical motions of white matter water molecules (Yamada et al., 2016), as well as for objectively predicting the post-surgery visual recovery of patients with PA. A strategy that combines OCT and DTI is increasingly used to predict visual impairment in degenerative neuropathy (Hubers et al., 2016; Alves et al., 2018) and visual pathways characteristic of amblyopia (Altintas et al., 2017). In our previous study, fractional anisotropy (FA) values of the visual pathway were found to be positively correlated with the thickness of the circumpapillary retinal nerve fiber layer (CP-RNFL) in patients with PA, suggesting the feasibility of combining OCT and DTI in evaluating the impairment of the entire visual pathway (Pang et al., 2022). The current study compared retinal OCT parameters and optic nerve DTI parameters in 53 patients with NFPA (classified into mild and heavy optic chiasma compression subgroups). The degree of adverse changes in OCT and DTI parameters was found to be stronger in the severe compression group than that in the mild compression group, suggesting the potential application of these parameters in assessing optic nerve damage in patients with NFPA.

## Materials and methods

### Patients

We conducted a retrospective clinical study involving 57 patients who were first diagnosed with NFPA and received tumor resection by endoscopic sphenoidal sinus surgery at the Affiliated Hospital of Guangdong Medical University from January 2020 to April 2022. All surgeries were done by the same experienced neurosurgeon and reasonable optic apparatus decompression was accomplished following tumor removal. Intraoperatively, the structure of the ultra care was paid to preserve relevant sellar/suprasellar neurovascular structures, and achieve adequate hemostasis to avoid postoperative compressing hematoma. Overfilling with skull base reconstructive materials was avoided to prevent optic apparatus compression. Fifty-three healthy subjects with matching average gender and age as the controls. All subjects underwent examination of best-corrected visual acuity (BCVA) and the visual field, OCT of the optical disk and macular, and DTI of the visual pathway.

The inclusion and exclusion criteria for the NFPA group were as follows. I. PA was indicated by plain MRI and enhanced examination of the brain. II. PA was the first complete resection obtained by endoscopic sphenoidal sinus surgery by the same brain surgeon without additional optic nerve damage and was confirmed by histopathological examination. III. NFPA was clinically diagnosed in patients aged between 18 and 60 years. IV. Non-contact intraocular pressure was ≤21 mmHg (1 mmHg = 0.133 kPa). V. There was no previous history of intracranial diseases and trauma, intracranial surgery, ocular trauma, glaucoma, neuroretinal disease, or internal eye surgery. VI. Previous refractive errors were <±6.0D (spherical mirror) and <3.00D (column mirror). VII. The OCT images were clear, and the DTI images were of good quality.

The inclusion and exclusion criteria for the control group were as follows. I. Non-contact intraocular pressure was ≤21 mmHg. II. Visual acuity or corrected visual acuity was ≥ 0.6, and refractive errors were <± 6.0D (spherical mirror) and <3.00D (column mirror). III. There was no previous history of intracranial diseases, trauma, or intracranial surgery. IV. There was no history of ocular trauma, glaucoma, neuroretinal diseases, or internal eye surgery. V. The subjects’ age and sex-matched those of the NFPA group. VII. The OCT image was clear, and the DTI images were of good quality.

This study was conducted in accordance with the principles of the Helsinki Declaration and was approved by the Ethics Committee of the Affiliated Hospital of Guangdong Medical University (Approval Document No. PJ2020-006 and VJ2020-006-03). All subjects signed informed consent forms.

### Visual field examination

The patients with NFPA underwent a visual field examination after a corrected refractive error (KowaAP7000 precision visual field meter, Kowa, Japan) before pituitary tumor resection. The visual field test was the center 30°. If solid vision disappeared and false-negative or false-positive errors exceeded 20%, the test was considered unreliable and hence repeated. Two reliable visual field examinations were performed for each patient, and mean defect (MD) was used to assess the overall visual field defect.

### Magnetic resonance examination of the tumor

**FIGURE 1:**
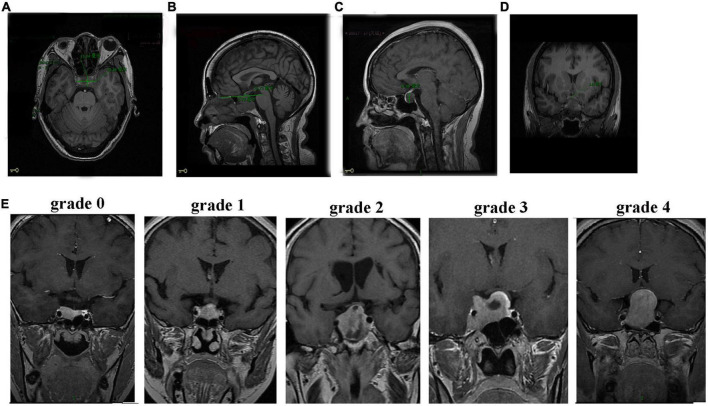
MRI images of patients with NFPA. **(A)** Axial position measurement of the horizontal diameter line (30.58 mm) and the anteroposterior diameter line (15.04 mm). **(B)** Sagittal measurements of the epitaxial height of the sella turcica diameter line (3.79 mm). **(C)** Sagittal measurements of the vertical diameter line (14.99 mm). **(D)** Thickness measurement of the optic chiasm. **(E)** Grades (0–4) of tumor compression on the optic chiasm.

All patients were subjected to a preoperative head MRI scan plus enhancement (Discovery MR750 3.0T, GE, USA) to measure their tumor sizes. The horizontal diameter line, anteroposterior diameter line, the epitaxial height of the sella turcica diameter line, the vertical diameter line of the tumor, and the thickness of the optic chiasma were recorded (Figures 1A–D). The maximum height of the vertical diameter line was measured in the sagittal view and used to determine the anteroposterior diameter line of the tumor. The epitaxial height of the sella turcica diameter line was measured as the height over the horizontal line between two points on the anterior and posterior tuberosity of the diaphragmatic saddle. The thickest position of the optic chiasma was selected for measurement. The tumor and optic chiasma were distinguished according to the degree of enhancement. Each value was measured three times and averaged. The degree of tumor compression on the optic chiasma was divided into five grades (Figure 1E). Grade 0 was defined as no contact between the tumor and optic chiasma. Grade 1 was defined as a tumor in contact with the optic chiasma but without surface deformation of the optic chiasma. Grade 2 was defined as a tumor in contact with the optic chiasma with surface deformation of the optic chiasma but visible suprachiasmatic cisterns. Grade 3 was defined as a tumor in contact with the optic chiasma, with the superior surface of the optic chiasma malformation and the superior chiasma cisterns invisible but without brain malformation. Grade 4 was defined as brain malformation, in addition to these changes. Four grade 0 patients who met the inclusion and exclusion criteria were excluded from this study because their tumors had no contact with the optic chiasma.

### OCT measures the thicknesses of RNFL, macular ganglion cell complex, inner plexus layer, and ganglion cell layer

The optic disk and macular area were scanned using 3D-OCT (Heideberg Engineering Spectralis, Germany). The OCT parameters included CP-RNFL of average thickness and four quadrants (nasal, supra, temporal, and inferior) and ganglion cell layer (GCL) and inner plexus layer (IPL) thicknesses of 1 mm macular center of the four quadrants (nasal, supra, temporal, and inferior) of the inner ring (1–3 mm from the macular fovea) and outer ring (within 3–6 mm from the macular fovea). Each parameter was evaluated automatically using Spectralis mapping software. GCC thickness was superimposed by the thicknesses of the macular RNFL, GCL, and IPL.

### DTI examination and image processing

**FIGURE 2:**
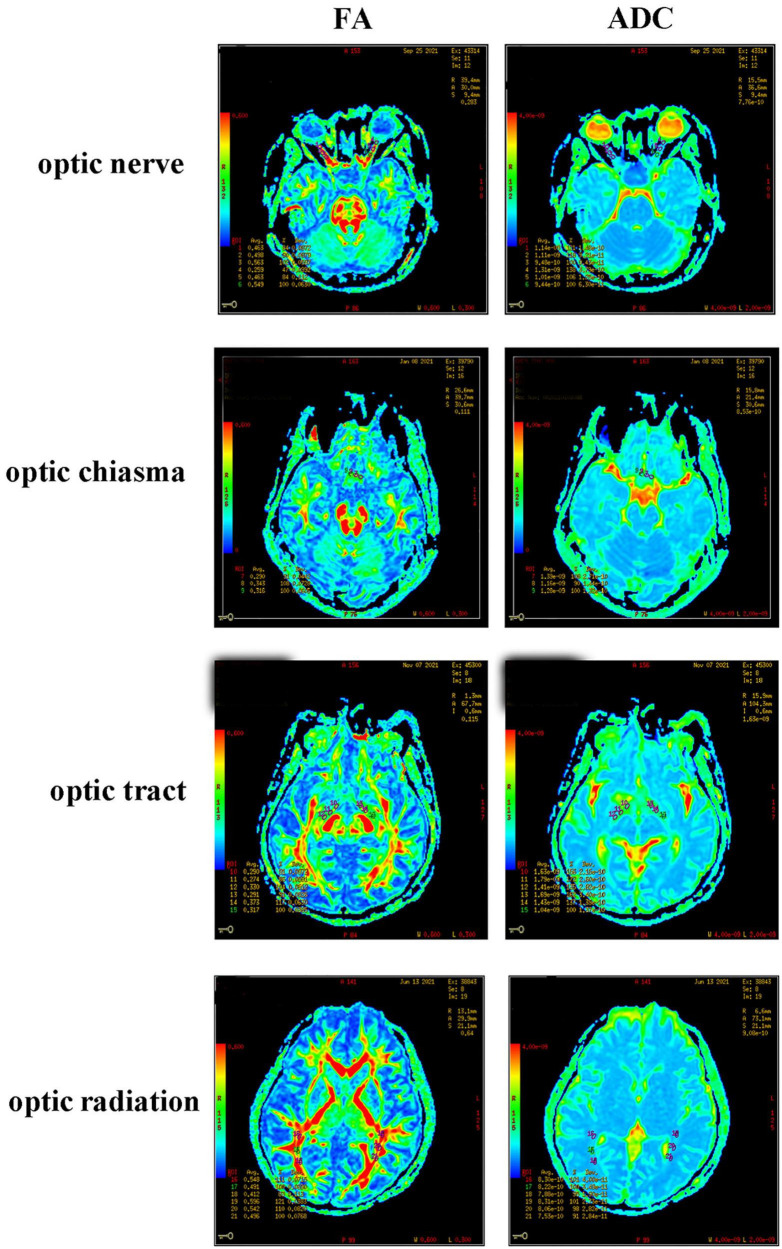
FA and ADC diagrams of the optic nerve, optic chiasma, optic tract, and optic radiation.

T1WI and DTI scanning were performed using a GE3.0T Optima MR360 imaging system with a head 16-channel phased-array coil. The T1WI scanning parameters were set as an axial 3D BRAVO sequence, 12.3/5.1 ms TR/TE, 256 × 256 matrix, 240 mm × 240 mm FOV, 1.4 mm layer thickness, 0 mm interval, and NEX 1. The DTI scanning parameters were set as a single-excitation DW-SE-EPI sequence, 9000/100.1 ms TR/TE, 128 × 128 matrix, 240 mm × 240 mm FOV, one acquisition, 25 diffusion-sensitive gradient directions, b value = 1000 s/mm^2^, layer thickness and layer spacing 2/0 mm, and axial scanning. The scanning results were presented in the form of a color-coded tensor FA graph and an ADC graph (Figure 2). In DTI data processing, these two graphs were set as green in the front and back directions, red in the left and right directions, and blue in the top and bottom directions. The anterior, middle, and posterior of the optic nerve, optic tract, and optic radiation, and the left, middle, and right of the optic chiasma were measured, analyzed, and recorded using the GE3.0 NMR machine software (ADW 4.2 Function Tool). Three regions of interest (ROIs) were selected at the clearest locations on the bilateral optic nerve, optic chiasma, bilateral optic tract, and optic radiation to measure the FA and ADC values. According to the classic neuroanatomical description and relevant literature (Li et al., 2019), the ROI was delineated and measured as an area of 8–12 mm^2^. In particular, when measuring an ultra-thin optic chiasm resulting from severe compression, FA and ADC signals were detected at the tumor edge, and the ROI became oval with a size of 8–12 mm^2^. To minimize measurement errors, image reconstruction and data measurement were performed by experienced physicians. The FA and ADC values of the optic nerve, optic chiasma, optic tract, and optic radiation were taken as the averages of the three ROIs.

### Statistical analysis

IBM SPSS24.0 statistical software was used for the statistical analysis, and the measurement data of normal distribution were expressed as mean ± standard deviation. An independent sample *t*-test was used for parameter comparison between groups, and a one-way ANOVA was used for parameter comparison among three groups and multiple groups, followed by a pairwise comparison conducted using the LSD-t test. Spearman correlation was used to evaluate the relationships among the best corrected visual acuity; visual field; RNFL, GCC, GCL, and IPL thicknesses; and the FA and ADC values of the DTI parameters. The area under the receiver operating characteristic (ROC) curve of the FA and ADC values was calculated and compared by conducting the *Z*-test to evaluate the diagnostic ability of the DTI parameters for optic path injury. To reduce the results bias, the statistical analysis of the data was performed by a dedicated researcher who was not selected from a group of radioactive data measurement researchers. A value of *P* < 0.05 was considered statistically significant.

## Results

### Subject groups and visual field comparison

The 53 patients were classified according to the degree of optic chiasma compression: grade 1 (13 patients), grade 2 (12 patients), grade 3 (11 patients), and grade 4 (17 patients). The average thicknesses of the optic chiasma in these four grades were (2.82 ± 0.98) mm, (2.62 ± 0.85) mm, (1.84 ± 0.73) mm, and (1.39 ± 0.78) mm, respectively, with a significant difference (*F* = 9.00, *P* < 0.05). Among these, there was a statistically significant difference between grades 1 and 2 and grades 3 and 4 but no statistically significant difference between grades 1 and 2 and between grades 3 and 4 (*P* = 0.01 for grade 1 vs. grade 3, *P* = 0.00 for grade 1 vs. grade 4, *P* = 0.03 for grade 2 vs. grade 3, *P* = 0.00 for grade 2 vs. grade 4, *P* = 0.55 for grade 1 vs. grade 2, and *P* = 0.18 for grade 3 vs. grade 4). Therefore, we combined grade 1 and grade 2 to form the mild optic chiasma compression group (case group 1) and grade 3 and grade 4 to form the heavy optic chiasma compression group (case group 2). The tumor body and optic chiasma in the two case groups were compared. The tumor suprasellar epitaxial height, anteroposterior diameter line, horizontal diameter line, and vertical diameter line in case group 2 were greater than those in case group 1, and the optic chiasma in case group 2 was thinner than that in case group 1 (Table 1).

**TABLE 1:** Visual acuity, visual field, and tumor were compared between mild and heavy optic chiasma compression case groups.

Tumor and optic chiasma parameters (mm)	Classification based on optic chiasma compression			*t*	*P*
	**Case group 1**	**Case group 2**	**Control group**		
BCVA (LogMAR)	0.15 ± 0.19	0.71 ± 0.61	0.03 ± 0.05		0.00
MD (dB)	1.29 ± 3.17	8.50 ± 7.53	0.62 ± 0.92		0.00
Epitaxial height of sella turcica diameter line	3.68 ± 2.76	16.69 ± 6.40		−9.39	0.00
Anteroposterior diameter line	19.98 ± 6.43	30.54 ± 7.91		−5.29	0.00
Horizontal diameter line	18.67 ± 6.43	26.57 ± 8.57		−3.78	0.00
Vertical diameter line	19.74 ± 6.10	36.08 ± 7.79		−8.42	0.00
Optic chiasma thickness	2.72 ± 0.91	1.57 ± 0.78	2.95 ± 0.25	4.97	0.00

The BCVA values of case group 1, case group 2, and the control group were (0.15 ± 0.19) LogMAR, (0.71 ± 0.61) LogMAR, and (0.03 ± 0.05) LogMAR, respectively, with significant differences (*F* = 79.58, *P* = 0.00). A comparison between the two groups showed *P* = 0.00 for case group 1 vs. case group 2, *P* = 0.03 for case group 1 vs. the control group, and *P* = 0.00 for case group 2 vs. the control group. The MD values of case group 1, case group 2, and the control group were (1.29 ± 3.17) dB, (8.50 ± 7.53) dB, and (0.62 ± 0.92) dB, respectively, with significant differences (*F* = 68.80, *P* = 0.00). A comparison between the two groups showed *P* = 0.00 for case group 1 vs. case group 2, *P* = 0.35 for case group 1 vs. the control group, and *P* = 0.00 for case group 2 vs. the control group.

### Comparison of CP-RNFL thickness among case group 1, case group 2, and the control group

The temporal CP-RNFL of case group 1 was significantly thinner than that of the control group (*P* = 0.04), and the CP-RNFL thickness of the other quadrants had no statistical significance between the two groups (*P*-values of average, nasal, supra, and inferior quadrants were 0.65, 0.56, 0.95, and 0.47, respectively) (Table 2). A comparison of CP-RNFL in all quadrants between case group 2 and the control group indicated statistically significant differences in thickness (all four quadrants had a *P*-value of 0.00) (Table 2).

**TABLE 2:** Comparison of CP-RNFL thickness between case group and control group.

CP-RNFL thickness (um)	Case group 1 (25 cases, 50 eyes)	Case group 2 (28 cases, 56 eyes)	Control group (53 cases, 106 eyes)	*F*	*P*
Average CP-RNFL	105.50 ± 15.82	81.55 ± 26.55	106.60 ± 11.65	40.27	0.00
Nasal CP-RNFL	73.60 ± 18.34	56.76 ± 26.26	71.72 ± 13.01	14.60	0.00
Supra CP-RNFL	131.68 ± 25.49	100.71 ± 34.56	131.61 ± 20.31	29.22	0.00
Temporal CP-RNFL	78.80 ± 17.30	59.83 ± 24.11	85.02 ± 13.79	36.80	0.00
Inferior CP-RNFL	136.16 ± 29.73	109.94 ± 35.87	139.76 ± 20.74	22.50	0.00

### Comparison of the thicknesses of GCC, IPL, and GCL among case group 1, case group 2, and the control group

Compared to the control group, case group 1 had significantly thinner GCC, IPL, and GCL within the macular center of 1 mm, GCC in the inferior aspect of the inner ring, and GCC in the temporal aspect of the outer ring (*P* = 0.00, 0.00, 0.00, 0.00, and 0.01) (Table 3), indicating that mild optic chiasma compression resulted in local thinning of the GCC in the macular area.

**TABLE 3:** Comparison of the thickness of GCC, IPL, and GCL between two case groups and control group.

Macular GCC, IPL, and GCL thickness (um)	Case group 1 (50 eyes)	Case group 2 (56 eyes)	Control group (106 eyes)	*F*	*P*
Center (1 mm diameter)	33.46 ± 9.79	34.48 ± 12.88	46.21 ± 10.13	34.05	0.00
	13.50 ± 5.16	15.04 ± 4.70	18.81 ± 4.39	27.88	0.00
	8.84 ± 4.73	8.94 ± 6.02	15.23 ± 4.74	44.53	0.00
Nasal-macular inner ring	105.79 ± 14.19	88.27 ± 22.54	107.93 ± 13.69	26.57	0.00
	39.26 ± 6.36	34.04 ± 9.43	39.81 ± 4.97	13.59	0.00
	46.50 ± 9.14	37.16 ± 13.10	46.74 ± 7.67	20.64	0.00
Supra-macular inner ring	111.89 ± 15.70	95.87 ± 23.57	113.04 ± 10.15	22.06	0.00
	38.91 ± 5.42	35.17 ± 8.70	39.33 ± 4.77	8.35	0.00
	50.17 ± 9.13	40.66 ± 13.25	49.90 ± 5.77	22.93	0.00
Temporal-macular inner ring	97.24 ± 14.52	89.37 ± 19.10	100.85 ± 9.08	12.22	0.00
	37.51 ± 6.20	34.04 ± 7.02	38.50 ± 4.55	10.55	0.00
	42.82 ± 8.90	37.16 ± 10.21	44.13 ± 5.59	15.29	0.00
Inferior-macular inner ring	104.67 ± 16.72	95.50 ± 20.76	111.55 ± 11.78	18.09	0.00
	37.82 ± 5.69	33.60 ± 7.98	38.43 ± 4.27	12.56	0.00
	45.75 ± 9.96	40.35 ± 10.17	48.05 ± 6.34	15.81	0.00
Nasal-macular outer ring	113.08 ± 15.55	99.09 ± 19.26	116.90 ± 10.75	26.45	0.00
	31.10 ± 4.32	26.52 ± 4.96	31.81 ± 3.50	28.66	0.00
	38.86 ± 8.10	32.91 ± 7.54	39.96 ± 5.78	20.49	0.00
Supra-macular outer ring	98.15 ± 14.82	90.55 ± 17.07	102.37 ± 11.02	12.78	0.00
	28.69 ± 3.63	26.69 ± 5.40	29.63 ± 3.32	9.26	0.00
	34.36 ± 7.48	31.66 ± 7.23	35.60 ± 5.20	7.23	0.00
Temporal-macular outer ring	84.08 ± 13.50	83.11 ± 12.48	89.19 ± 8.45	6.77	0.00
	31.76 ± 3.80	30.95 ± 5.37	32.79 ± 3.70	3.55	0.03
	35.37 ± 6.67	33.39 ± 7.59	36.18 ± 5.18	3.78	0.02
Inferior-macular outer ring	98.55 ± 14.28	92.16 ± 15.32	99.45 ± 12.87	5.10	0.01
	27.96 ± 4.08	26.13 ± 5.07	28.41 ± 3.79	4.99	0.00
	34.34 ± 5.93	31.10 ± 6.57	34.28 ± 8.26	4.02	0.02

Compared to the control group, case group 2 had significantly thinner GCC, IPL, and GCL in the macular center of 1 mm; nasal-, supra-, temporal-, and inferior-macular inner rings; and nasal-, supra-, temporal-, and inferior-macular outer rings (all had *P*-values < 0.05) (Table 3), indicating that the GCC in the macular area was thinner diffusely with increasing compression of the optic chiasma.

### Comparison of the FA and ADC values among case group 1, case group 2, and the control group

A comparison of case group 1 with the control group showed that the FA values of the optic nerve, optic chiasma, and optic tract in case group 1 decreased significantly (*P* = 0.00, 0.01, and 0.00), while the ADC values of the optic nerve and optic chiasma increased significantly (*P* = 0.01 and 0.04) (Table 4). A comparison of case group 2 with the control group showed that the FA values of the optic nerve, optic chiasma, optic tract, and optic radiation in case group 2 decreased significantly (*P* = 0.00, 0.00, 0.00, and 0.03), while the ADC values of the optic nerve, optic chiasma, and optic radiation increased significantly (*P* = 0.00, 0.00, and 0.00) (Table 4). There was no statistical difference in the ADC values of the optic tracts in group 1 and group 2 compared to the control group (*P* = 0.55 and 0.24) (Table 4).

**TABLE 4:** Comparison of the FA value and ADC value between the two case groups and the control group.

FA value and ADC value (×10^–9^mm^2^/s)	Case group 1 (50 eyes)	Case group 2 (56 eyes)	Control groups (106 eyes)	*F*	*P*
Optic nerve	0.38 ± 0.09	0.35 ± 0.11	0.43 ± 0.10	13.83	0.00
	1.53 ± 0.31	1.64 ± 0.39	1.38 ± 0.27	12.53	0.00
Optic chiasma	0.28 ± 0.08	0.20 ± 0.07	0.32 ± 0.05	30.37	0.00
	1.86 ± 0.51	2.02 ± 0.37	1.66 ± 0.39	5.04	0.01
Optic tract	0.42 ± 0.10	0.34 ± 0.10	0.48 ± 0.09	39.16	0.00
	1.22 ± 0.35	1.25 ± 0.33	1.18 ± 0.31	0.72	0.49
Optic radiation	0.53 ± 0.05	0.49 ± 0.07	0.52 ± 0.05	4.90	0.01
	0.86 ± 0.06	0.88 ± 0.07	0.85 ± 0.06	4.25	0.00

### Analysis of the correlation of BCVA and MD values with DTI and OCT parameters

We performed a correlation analysis between the BCVA and MD values and all DTI and OCT parameters and displayed the parameters with the strongest correlation with BCVA and MD values. The BCVAlogMAR and MD values showed the strongest negative correlation with the FA value of the optic chiasma (*r* = −0.51, −0.54, *P* = 0.00), and BCVAlogMAR showed the strongest negative correlation with the superior CP-RNFL thickness (*r* = −0.53, *P* = 0.00) (Table 5). The MD value of the visual field showed the strongest negative correlation with CP-RNFL thickness from the supra and average aspects, respectively (both *r* = –0.62, *P* = 0.00) (Table 5). BCVAlogMAR had the strongest positive correlation with the tumor’s suprasellar epitaxial height (*r* = 0.44, *P* = 0.00). The MD value had the strongest positive correlation with the vertical diameter of the tumor (*r* = 0.60, *P* = 0.00) (Table 5). These data suggest that the degree of tumor compression on the vertical diameter line on the optic chiasma indicates the degree of visual field impairment.

**TABLE 5:** Correlation analysis between BCVAlogMAR and MD and DTI parameters and OCT parameters.

Correlation coefficient	BCVAlogMAR	MD
**Optic nerve FA**		
r	-0.44	-0.50
*P*	0.00	0.00
**Optic chiasma FA**		
r	-0.51	-0.54
*P*	0.00	0.00
**Optic tract FA**		
r	-0.41	-0.44
*P*	0.00	0.00
**Optic radiation FA**		
r	-0.36	-0.41
*P*	0.01	0.00
CP-RNFL	Supra CP-RNFL	Supra or Average CP-RNFL
r	-0.53	-0.62
*P*	0.00	0.00
Macular IPL	Inferior outer ring	Supra inner ring
r	-0.45	-0.43
*P*	0.00	0.00
Macular GCL	Supra inner ring	Supra inner ring
r	-0.37	-0.53
*P*	0.01	0.00
Macular GCC	Nasal inner ring	Supra inner ring
r	-0.38	-0.51
*P*	0.01	0.00
PA diameter	Epitaxial height of suprasellar extension	Vertical diameter
r	0.44	0.60
*P*	0.00	0.00
**Optic chiasma thickness**		
r	-0.47	-0.46
*P*	0.00	0.00

### Correlation between DTI and OCT parameters and the tumor diameter line

In all patients with NFPA, the correlation of FA and ADC values of the optic nerve, optic chiasma, optic tract, and optic radiation with RNFL thickness, macular IPL, macular GCL, and macular GCC was analyzed, and the strongest correlation was displayed. As shown in Table 6, the strongest positive correlations were observed between the FA value of the optic nerve and average CP-RNFL thickness (*r* = 0.58, *P* = 0.00), between the FA value of the optic chiasma and thickness of IPL and GCC in the inferior-macular inner ring (*r* = 0.52, *P* = 0.00), between the FA value of the optic tract and inferior CP-RNFL thickness (*r* = 0.47, *P* = 0.00), and between the FA value of optic radiation and superior CP-RNFL thickness (*r* = 0.46, *P* = 0.00) (Table 6). These data suggest that the average CP-RNFL thickness, and thickness of inferior-macular inner-ring IPL, GCC, inferior CP-RNFL, and superior CP-RNFL are the best indicators of the impairment of the optic nerve, optic chiasma, optic tract, and optic radiation, respectively.

**TABLE 6:** The strongest correlation between DTI parameters and OCT parameters.

Visual pathway DTI parameters	CP-RNFL thickness	Macular GCC thickness	Macular IPL thickness	Macular GCL thickness	Diameter line of tumor
Optic nerve FA	Average	Center (1 mm diameter)	Center (1 mm diameter)	Center (1 mm diameter)	Vertical diameter line
r	0.58	0.43	0.51	0.43	−0.38
*P*	0.00*	0.00*	0.00*	0.00*	0.01*
Optic chiasma FA	Average	Inferior-macular inner ring	Inferior-macular inner ring	Inferior-macular inner ring	Epitaxial height of sella turcica diameter line
r	0.44	0.52	0.52	0.48	−0.62
*P*	0.00*	0.00*	0.00*	0.00*	0.00*
Optic tract FA	Inferior	Nasal-macular outer ring	Nasal-macular outer ring	Center (1 mm diameter)	Vertical diameter line
r	0.47	0.40	0.41	0.40	−0.39
*P*	0.00*	0.00*	0.00*	0.00*	0.01*
Optic radiation FA	Supra	Temporal-macular outer ring	Nasal-macular outer ring	Temporal-macular outer ring	Horizontal diameter line
r	0.46	0.38	0.45	0.41	−0.43
*P*	0.00*	0.00*	0.00*	0.00*	0.00*

*P < 0.001.

The strongest negative correlations were observed between the FA value of the optic nerve and optic tract and the tumor vertical diameter line (*r* = −0.38, *P* = 0.01; *r* = −0.39, *P* = 0.01), between the FA value of the optic chiasma and epitaxial height of the tumor suprasellar (*r* = −0.62, *P* = 0.00), and between the FA value of the optic radiation and the tumor horizontal diameter line (*r* = −0.43, *P* = 0.00) (Table 6). These data suggest that the degree of tumor compression on the vertical diameter line to the optic chiasma indicates impaired nerve fiber conduction.

### Comparison of the test effectiveness of the FA and ADC values in visual pathway damage

**FIGURE 3:**
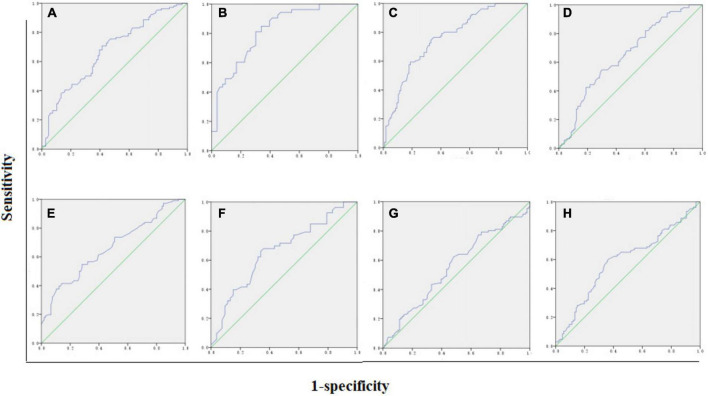
AUC of FA and ADC values. **(A)** Optic nerve FA = 0.68. **(B)** Optic chiasma FA = 0.82. **(C)** Optic tract FA = 0.76. **(D)** Optic radiation ADC = 0.65. **(E)** Optic nerve ADC = 0.66. **(F)** Optic chiasma ADC = 0.66. **(G)** Optic tract ADC = 0.55. **(H)** Optic radiation ADC = 0.60.

To evaluate the diagnostic ability of the FA and ADC values for damage to the optic nerve, optic chiasma, optic tract, and optic radiation, we calculated the area under the curve (AUC) on the ROC curve of these values. The Z value of the AUC on the ROC curve of the FA and ADC values of the structures of the entire visual pathway showed that the Z optic nerve = 0.71 (*P* = 0.48), Z chiasma = 3.77 (*P* = 0.00), Z optic tract = 4.95 (*P* = 0.00), and Z visual radiation = 1.18 (*P* = 0.23) (Figure 3), indicating that the FA values of the visual chiasma and optic tract were more effective than the ADC values.

## Discussion

Retinal RNFL can be evaluated from the changes in two substructures, nasal crossed fibers and temporal uncrossed fibers, which are bounded by the vertical line of the fovea. In our study, patients with NFPA showed early thinning of the temporal CP-RNFL quadrant, the quadrant in which the papillary macular fibrosis was located. According to the literature, the temporal CP-RNFL thickness is correlated with visual function. For example, Kawaguchi et al. (2019) found that the thickness of the temporal CP-RNFL was significantly correlated with visual function recovery, and the thinning of the temporal nerve fiber layer had the greatest impact on vision because the papillomacular fibers converged from the temporal side of the optic disk. Glebauskiene et al. (2018) found that the thickness of the temporal CP-RNFL was positively correlated with the distance between the optic chiasma and the pituitary tumor. However, none of these studies revealed a relationship between changes in CP-RNFL thickness and the degree of chiasmatic compression. In our study, mild optic chiasma compression showed thinning of the temporal CP-RNFL, while heavy optic chiasma compression showed thinning of the CP-RNFL in all quadrants, indicating the progression of the CP-RNFL from temporal lesions to diffuse atrophy in patients with NFPA.

Agarwal et al. (2021) suggested that GCL-IPL had a stronger diagnostic ability for optic nerve injury than CP-RNFL. In addition, Zhang et al. (2019) found that the GCL was thinned in patients with early PA without visual field defects. Moon analyzed the OCT multilayer structure of the retinas of patients with PA and found that the thicknesses of both GCL and IPL decreased as compared with the control (Moon and Shin, 2020). Sun et al. (2017) indicated that the GCC thickness in patients with PA presented quadrant characteristics, mainly nasal-macular thinning. Consistent with these studies, we found that the GCC, GCL, and IPL in the macular area of NFPA patients with mild optic chiasma compression were damaged locally. Furthermore, heavy optic chiasma compression caused diffuse thinning of the GCC, IPL, and GCL, indicating that the tumor compresses both crossed and non-crossed nerve fibers with the disease progression. Based on this, we infer that similar to the case of CP-RNFL, thinning of the GCC also starts from a single quadrant and eventually extends to the entire macular region.

The feasibility of OCT indicators in predicting the reversibility of visual impairment remains controversial. Iqbal et al. (2020) found that postoperative visual acuity improved in patients with normal preoperative RNFL thickness but did not in those with preoperative RNFL thinning. An et al. indicated that GCC damage occurred before RNFL thinning (Micieli et al., 2019). However, Lukewich and Micieli (2019) argued that RNFL thinning appeared earlier than GCC thinning. One of the reasons for the controversy is that PA tumors affect the entire visual pathway (from the retina to the visual center of the brain), and the OCT indicators of the retina alone are inadequate for understanding the entire visual pathway impairment. Thus, it is necessary to recognize the objective evaluation effect of DTI parameters on visual impairment. Decreased FA values and increased ADC values arise with the loss of nerve fibers and atrophy of nerve tissue (Lee et al., 2005). Anik et al. (2011) found that a lower preoperative optic nerve FA value and higher mean defect MD value were correlated with poor visual effect at 6 months in patients with PA, and determined DTI parameters to be a good predictor for PA surgery (Anik et al., 2018). Our data showed decreased FA value and increased ADC value in parts of the visual pathway of NFPA patients with mild and heavy optic chiasma compression, indicating that even mild compression of the optic chiasma results in impaired signal transduction. The major contribution of this study was that the decrease in the FA value and the increase in the ADC value were synchronized with quadrant thinning of the CP-RNFL, GCC, GCL, and IPL. This optic nerve appearance indicates that the conduction function and structural abnormalities of nerve fibers are present simultaneously in patients with NFPA. In previous studies, in patients with early PA, CP-RNFL thickening and GCC thinning appeared earlier than visual field defects, while nerve fiber functional impairment appeared later than structural abnormalities (Tieger et al., 2017; Blanch et al., 2018; Monteiro, 2018; Micieli et al., 2019). However, some contrasting findings have also been noted. For example, Lukewich and Micieli (2019) found visual field abnormalities to appear earlier than OCT GCC and CP-RNFL thinning. However, the combination of DTI and OCT results in the present study showed that the functional and structural abnormalities of optic nerve fibers in patients with NFPA appeared simultaneously.

Our exploration of the posterior optic pathway indicated that, as compared to the control group, ADC values did not change in the mild chiasma compression group but increased significantly in the heavy chiasma compression group. However, the ADC values of the optic tract did not change significantly. These results are in agreement with those of John et al., who also found no statistical difference in the MD values of the optic tract in the control group (Rutland et al., 2019). In a previous study, increased MD or ADC values reflected reduced limits on water diffusion due to myelin or axon membrane damage (Lutz et al., 2008). Is there a different mechanism through which the ADC values of the optic tract change? At present, there are a few similar studies, and the specific mechanism needs to be further explored.

We conducted an AUC calculation of the FA and ADC values on the ROC curve of visual pathway injury and found that the area under the ROC curve of the FA value of the optic chiasma and the optic tract was larger than that of the ADC value, and the difference was statistically significant. In particular, the area under the ROC curve of the FA value of the optic chiasma was the largest, indicating the best performance for the diagnosis of visual pathway impairment. Although no study has been conducted on the diagnostic ability of DTI parameters for visual pathway injury in patients with NFPA, according to a previous comparison of FA and MD values for visual pathway injury in glaucoma patients, both FA values of the optic nerve and those of optic radiation showed high sensitivity and specificity (Sidek et al., 2014). The reason why the FA value is better than the ADC value may be that the ADC value is affected by complex factors.

This study also has some limitations. First, as the imaging principle of DTI is an echo sequence that is more sensitive to motion, motion artifacts are inevitable. Second, artificial measurement errors are inevitable due to several factors (i.e., the complex distribution of white matter fibers, long visual pathways, and multiple cross fibers, especially in the optic chiasma, and the complex position relationship between PA and optic chiasma). Third, DTI detection is susceptible to interference factors (e.g., skull and gas, hemorrhage artifacts, old brain injury, and primary encephalopathy). All these factors need to be addressed in the future.

In conclusion, the DTI parameter FA and OCT parameters RNFL and macular GCC, GCL, and IPL can be used to evaluate visual pathway impairment in patients with NFPA. In particular, the FA value of the optic chiasma has a high diagnostic ability for visual pathway impairment. The coupled DTI and OCT can be used to comprehensively understand the microscopic changes in the structure and function of the visual pathway and to more objectively evaluate the visual pathway damage induced by PA. We further explored the predictive ability of this preoperative assessment for postoperative visual recovery.

## Data availability statement

The original contributions presented in this study are included in the article/supplementary material, further inquiries can be directed to the corresponding authors.

## Ethics statement

The studies involving human participants were reviewed and approved by Ethics Committee of the Affiliated Hospital of Guangdong Medical University. The patients/participants provided their written informed consent to participate in this study.

## Author contributions

YHP, ZT, and JXZ contributed to the conception and design of the work. XXC, ZHL, XY, and QZ were responsible for the acquisition and analysis of data. BQH, WM, and QSZ interpreted the data. YHP drafted the work. WM substantively revised it. All authors reviewed the manuscript and approved the submitted version.
